# Escalating to medium‐ versus high‐efficacy disease modifying therapy after low‐efficacy treatment in relapsing remitting multiple sclerosis

**DOI:** 10.1002/brb3.3498

**Published:** 2024-04-30

**Authors:** Jannis Müller, Izanne Roos, Tomas Kalincik, Johannes Lorscheider, Edoardo Galli, Pascal Benkert, Sabine Schädelin, Sifat Sharmin, Maximilian Einsiedler, Peter Hänni, Jürg Schmid, Jens Kuhle, Tobias Derfuss, Cristina Granziera, Tjalf Ziemssen, Timo Siepmann, Özgür Yaldizli

**Affiliations:** ^1^ Neurology Clinic and Policlinic, Departments of Head, Spine and Neuromedicine, MS Center and Translational Imaging in Neurology (ThINk) Basel, Department of Biomedical Engineering, Research Center for Clinical Neuroimmunology and Neuroscience Basel (RC2NB) University Hospital Basel and University of Basel Basel Switzerland; ^2^ Division of Health Care Sciences Dresden International University Dresden Germany; ^3^ CORe, Department of Medicine University of Melbourne Melbourne Australia; ^4^ Neuroimmunology Centre Royal Melbourne Hospital Melbourne Australia; ^5^ Department of Clinical Research Clinical Trial Unit University Hospital Basel Basel Switzerland; ^6^ Swiss Federation for Common Tasks of Health Insurances (SVK) Solothurn Switzerland; ^7^ Department of Neurology, Medical Faculty and University Hospital Carl Gustav Carus Technische Universität Dresden Dresden Germany; ^8^ Center of Clinical Neuroscience, Department of Neurology, Medical Faculty and University Hospital Carl Gustav Carus Technische Universität Dresden Dresden Germany

**Keywords:** disease modifying therapies, early‐intensive, escalation, treatment selection, treatment strategies

## Abstract

**Background:**

In patients with relapsing remitting multiple sclerosis (RRMS) on low‐efficacy disease modifying therapies (DMT), the optimal strategy on how to escalate treatment once needed, remains unknown.

**Methods:**

We studied RRMS patients on low‐efficacy DMTs listed in the Swiss National Treatment Registry, who underwent escalation to either medium‐ or high‐efficacy DMTs. Propensity score‐based matching was applied using 12 clinically relevant variables. Both groups were also separately matched with control subjects who did not escalate therapy. Time to relapse and to disability worsening were evaluated using Cox proportional hazard models.

**Results:**

Of 1037 eligible patients, we 1:1 matched 450 MS patients who switched from low‐efficacy to medium‐efficacy (*n *= 225; 76.0% females, aged 42.4 ± 9.9 years [mean ± SD], median EDSS 3.0 [IQR 2–4]) or high‐efficacy DMTs (*n *= 225; 72.4% females, aged 42.2 ± 10.6 years, median EDSS 3.0 [IQR 2–4]). Escalation to high‐efficacy DMTs was associated with lower hazards of relapses than medium‐efficacy DMTs (HR = 0.67, 95% CI 0.47–0.95, *p *= .027) or control subjects (HR = 0.61, 95% CI 0.44–0.84, *p = *.003). By contrast, escalation from low to medium‐efficacy DMTs did not alter the hazard for relapses when compared to controls (i.e. patients on low‐efficacy DMT who did not escalate DMT during follow‐up)

**Conclusion:**

Our nationwide registry analysis suggests that, once escalation from a low‐efficacy DMT is indicated, switching directly to a high‐efficacy treatment is superior to a stepwise escalation starting with a moderate‐efficacy treatment.

## Graphical abstract

This study investigated two escalation strategies in patients with multiple sclerosis receiving low‐efficacy treatment: Stepwise escalation to medium‐efficacy treatment vs. direct escalation to high‐efficacy treatment. It found that direct escalation to high‐efficacy therapy is superior in preventing future relapses. It therefore advocates for a direct escalation to high‐efficacy treatment once needed, in patients initially following an escalation approach by starting low‐efficacy treatment.

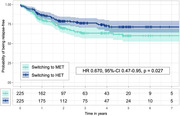

## 1 INTRODUCTION

To date, more than 15 disease modifying therapies (DMTs) are licensed for the treatment of relapsing remitting multiple sclerosis (RRMS), with different modes of action, routes of administration and adverse events (Hauser & Cree, 2020). This diversity presents both opportunities and challenges in clinical practice, as physicians must carefully balance the potential of effective disease control against tolerability, risks, and side effects (Inojosa et al., 2022). Still, navigating the uncertainty around switching or escalating treatment remains one of the most paramount responsibilities of MS care providers (Gross & Corboy, 2019). To facilitate individualized decision‐making, several treatment strategies have been firmly established, with two notable approaches taking the forefront: (Casanova et al., 2022; Filippi et al., 2022) (1) the *escalation* strategy, wherein patients start with low‐efficacy DMTs, and switch to a higher‐efficacy drug, once there is reason for treatment escalation, and (2) the *early‐intense* strategy, wherein patients start with a high‐efficacy therapy during early disease stages, potentially capitalizing on a so‐called window of opportunity. While the *escalation* strategy held dominance in the past, recent studies have indicated superior long‐term outcomes with the *early‐intensive* approach (Brown et al., 2019; Buron et al., 2020; Harding et al., 2019; He et al., 2020; Iaffaldano et al., 2021; Simonsen et al., 2021; Spelman et al., 2021). Nonetheless, until 2018, also owing to historical practice patterns, over 60% of treatment‐naive patients commenced treatment with low‐efficacy DMTs (Freeman et al., 2021; Henderson et al., 2023). Since then, this group transitioned to become the second largest group (after medium‐efficacy DMTs) (Freeman et al., 2021; Henderson et al., 2023). While many patients demonstrate excellent response to low‐efficacy DMTs over extended periods (Sormani et al., 2016), a considerable subset of patients may require an escalation from low‐ to higher‐efficacy DMTs at any time of their treatment trajectory, with disease breakthrough (Bsteh et al., 2021), early or delayed adverse events (Walther & Hohlfeld, 1999), or patient preference (Balak et al., 2013) being the most prominent reasons for escalation.

To date, evidence is scarce on guiding treatment escalation in these patients. Adhering to the *escalation* approach would involve progressing to the next level of effectiveness, while the emerging evidence favoring an *early‐intensive* strategy would advocate for the prompt implementation of high‐efficacy therapy. Hence, in the present work, we aim to contribute to this ongoing debate on the optimal treatment strategy, by comparing clinical outcomes of patients who initially received low‐efficacy treatments and subsequently escalated to either medium‐ or high‐efficacy therapies.

## 2 MATERIALS AND METHODS

### 2.1 Database

We used data from the Swiss national MS treatment registry, which is administered by the Swiss Federation for Common Tasks of Health Insurances (Schweizerischer Verband für Gemeinschafts‐aufgaben der Krankenversicherer, SVK). The SVK serves as an administrative repository for overseeing DMT reimbursements within Switzerland's mandatory health insurance system. Between 1995 and 2012, the SVK covered about 85% of the Swiss population. Following the departure of a large health insurer in 2013, the coverage subsequently remained stable at around 67% of the Swiss population (Swiss Federation for Common Tasks of Health Insurances, 2014).

As of April 2023, the database contained anonymized, patient‐level DMT reimbursement information from > 125,000 visits of > 18,000 MS patients. As part of the annual routine DMT reimbursement process, treating board‐certified neurologists prospectively reported standardized clinical data to the SVK (MS diagnosis and clinical phenotype, patients’ age, gender, date of disease onset, and once a year: date of last relapse, number of relapses in the prior year, and neurological status assessed by the Expanded Disability Status Scale [EDSS]). After rigorous screening for data coherence by an independent SVK reviewer, the data were anonymized and integrated into a secured database. Patients were followed up by the SVK until they discontinued DMT or changed their health insurance to a company that was not affiliated with the SVK. Compared to other Swiss population‐based studies of MS patients, the SVK database showed MORE secondary progressive MS patients, and more patients on first‐line injectables (Kaufmann et al., 2019). Differences between cohorts may be attributed to specific cohort inclusion criteria, recruitment timing and cohort settings (for further information, refer to Supplementary Text 3).

### 2.2 Inclusion and exclusion criteria

We included data collected after May 1, 2007 from patients who met the following inclusion criteria: (1) diagnosis of clinically definite RRMS (Poser et al., 1983), (2) age ≥18 years, (3) minimal data set (i.e., sex, year of birth, date of MS onset, for each visit: last relapse), (4) minimal follow‐up (defined as ≥2 EDSS assessment prior, and ≥2 after baseline), and for the primary analysis, (5) treatment with low‐efficacy DMTs for ≥2 years and subsequent escalation to (a) medium‐ or (b) high‐efficacy therapy.

We excluded patients with (1) progressive MS forms, (2) clinically isolated syndrome, and (3) treatment with alemtuzumab, cladribine, daclizumab, mitoxantrone, or stem cell transplantation at any time during follow‐up.

### 2.3 Study design and outcomes

This study was designed as a retrospective analysis of prospectively collected data, comparing clinical outcomes of patients on low‐efficacy DMT undergoing treatment escalation to either medium‐ or high‐efficacy DMT, with the null hypotheses stating that there are no differences in clinical outcomes between the groups. To identify treatment escalators, we classified the DMTs into three groups of efficacy: (Hauser & Cree, 2020) (A) low‐efficacy treatment (LET), including interferone beta‐1a, peginterferon beta‐1a, interferone beta‐1b, glatiramer acetate, teriflunomide; (B) medium‐efficacy treatment (MET), encompassing fingolimod, dimethyl fumarate, ponesimod, ozanimod; and (C) high‐efficacy treatment (HET), namely ocrelizumab and natalizumab.

### 2.4 Primary and secondary analyses (Figure 1)

In the primary analysis, we compared patients who switched from LET to MET (i.e., from A to B) with patients who switched from LET to HET (i.e., A to C). We employed a “per protocol” approach, only considering outcome events occurring on the drug after escalation, with censoring at treatment switch or end of follow‐up, whichever came first. On secondary analyses, we compared each of the escalating groups separately with patients who did not change their treatment and remained on low‐efficacy DMTs (“controls”). In all scenarios, the baseline was established using the date of the initial reimbursement for the drug that was intended to be given after the escalation.

### 2.5 Outcomes

The following clinical outcomes were used: (1) relapses, defined as a new or exacerbating neurological symptom that persisted for ≥24 h, in absence of concurrent fever, that appeared ≥30 days after a previous relapse (Confavreux et al., 2000). Additionally, we reported the annualized relapse rate (ARR) after escalation for each patient, calculated as the total number of relapses divided by the years of follow‐up until censoring (2) 12‐month‐confirmed EDSS worsening in absence of a reported relapse in the previous year, defined in a stepwise stratified manner, as an increase of ≥1.5 EDSS points, if baseline EDSS was 0, ≥1 points if baseline EDSS was 1.0–5.5, and ≥0.5 points if baseline EDSS was ≥6 (Müller et al., 2023).

### 2.6 Sensitivity analyses

We additionally compared the escalating groups when exclusively considering patients (1) with or (2) without a relapse in the year prior to escalation, and (3) when applying an “intention to treat” approach, focusing on outcome events occurring during the entire postescalation follow‐up, irrespective of whether the patients were still on the escalated DMT or not.

### 2.7 Matching and statistical analysis

All analyses were performed in R (Version 4.2.0). Data quality control procedures applied prior to patient inclusion are summarized in Supplementary Text 1.

#### 2.7.1 Matching

To balance the groups for their baseline characteristics, the propensity of treatment escalation strategy was estimated using a multivariable logistic regression model with treatment allocation as outcome and age, sex, disease duration, EDSS, previous treatment, duration on the previous treatment, number of previous treatments, number of relapses in the prior 1 and 2 years, total number of prior relapses, and time since last relapse as independent variables. We employed nearest neighbor matching in a 1:1 ratio, with a caliper of 0.2 standard deviations (SD) of the propensity score, without replacement.

**FIGURE 1:**
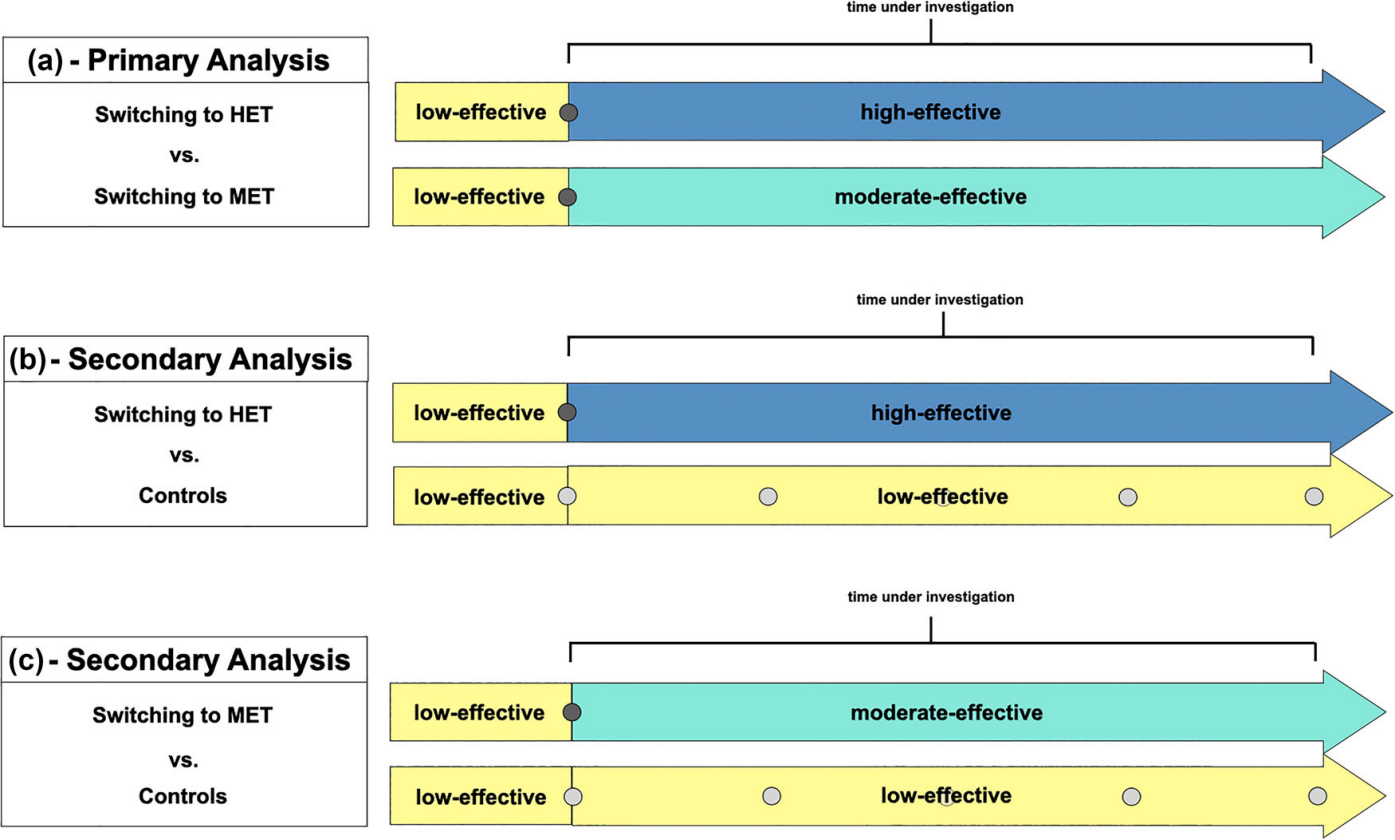
Study design of different analyses. (a) Comparison of patients switching to MET versus patients switching to HET. (b) Comparison of patients escalating to HET versus controls. (c) Patients escalating to MET versus controls. Dark gray dots indicate timepoints of matching. Bright gray dots indicate possible timepoints of matching. HET, high‐efficacy treatment, MET, medium‐efficacy treatment.

**FIGURE 2:**
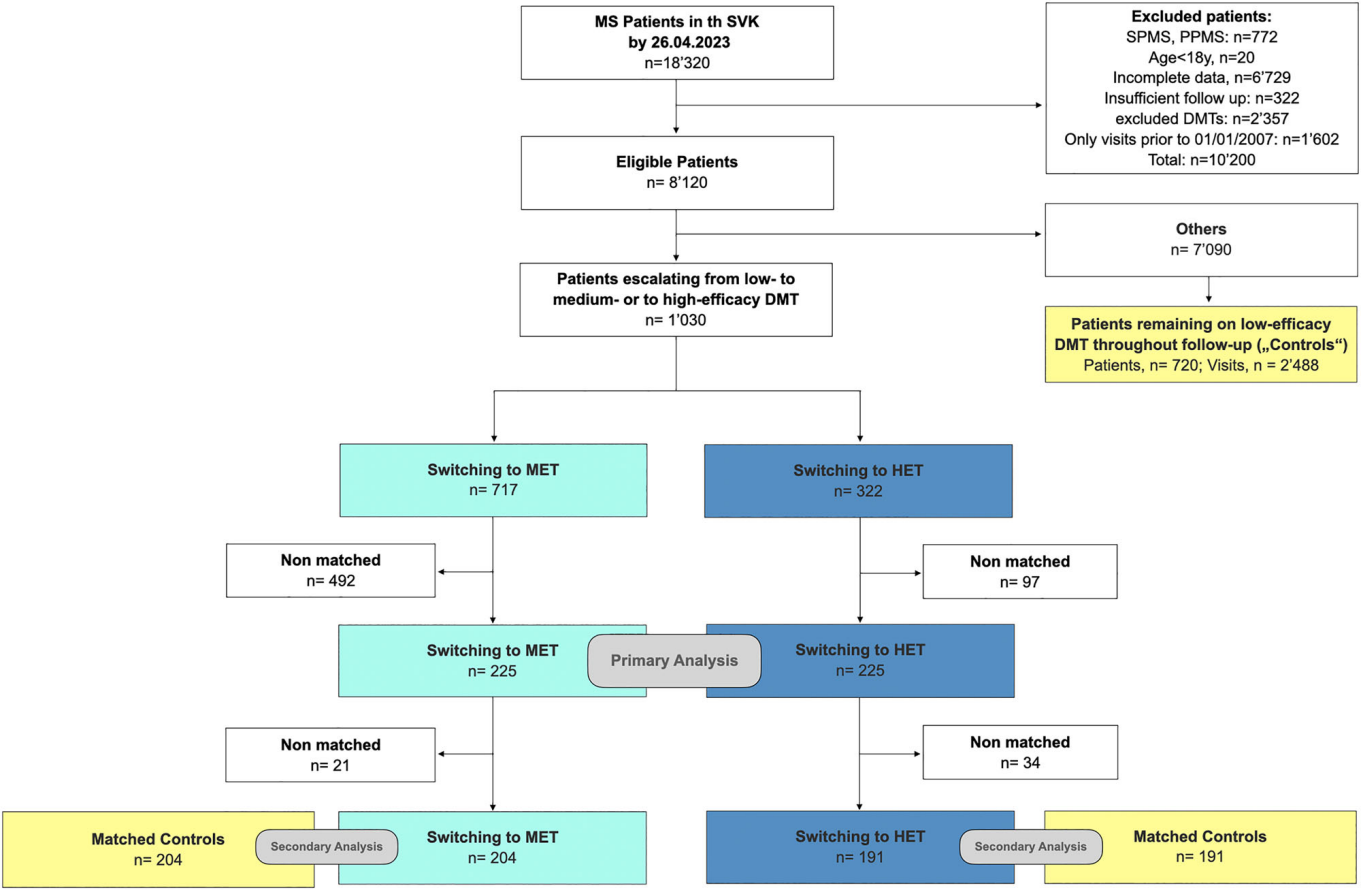
Low diagram of patients included in the study, i.e. patients switching from LET to MET (turquoise), patients switching from LET to HET (dark bue) and controls (yellow). DMT, disease modifying therapy; HET, high‐efficacy treatment; *n*, number; MET, medium‐efficacy treatment; PPMS, primary progressive multiple sclerosis; SPMS, secondary progressive multiple sclerosis; SVK, Schweizerischer Verband für Gemeinschaftsaufgaben der Krankenversicherer (Swiss Federation for Common Tasks of Health Insurances).

Using this methodology, we first matched patients who switched from LET to MET versus from LET to HET. Subsequently, each of these groups was matched separately with the controls (Figures 1 and 2). To facilitate the latter, any documented visit of the controls that fulfilled the inclusion criteria I‐IV (see above) could serve as potential matching timepoint, a methodology previously applied by Brown et al. (2019). This strategy was chosen to accommodate the formal absence of a switching date in the controls, allowing us to find the most suitable matching partner for each escalating patient. This included the possibility that one control subject could be matched multiple times during her/his trajectory. Balance after matching was assessed using standardized mean differences (SMD), and a SMD < 0.2 was considered as sign of adequate balance (Andrade, 2020).

#### 2.7.2 Statistical analysis

Clinical outcomes were explored using Kaplan–Meier plots and analyzed via conditional proportional hazard models (Cox) to assess the likelihood of remaining free from relapses or EDSS worsening, respectively. In these models, we used a cluster term to indicate the matching pair, and an inverse weighting for individual controls who were matched multiple times during their available follow‐up (secondary analysEs). Censoring was performed at the last documented visit in the SVK, which encompassed treatment termination, departure from the SVK, loss to follow‐up, or death, amongst others. We applied pair‐wise censoring, with the joint follow‐up time determined as the shorter of the two individual follow‐up periods for each matched patient‐pair. The proportional hazards assumption was assessed by calculating the Schoenfeld's global test. The ARR was compared between the groups using negative binomial models, with the matching pair as cluster term and the time to censoring as covariate.

Results were reported as hazard ratios (HR) and rate ratios (RR), respectively, as well as with 95% confidence intervals (CI) and *p*‐values. A two‐sided *p ≤*.05 was considered as statistically significant. Because there was no adjustment for multiple comparisons, secondary analyses should be interpreted as exploratory. Continuous data were given as mean ± standard deviation (SD), discrete and ordinal variables as median and interquartile range (IQR). Categorical data were summarized using counts and percentages.

## 3 RESULTS

### 3.1 Cohort description

The inclusion criteria yielded a total of 1037 escalating patients. Demographics and clinical characteristics before and after matching are shown in Table 1.

**TABLE 1:** Characteristics of patients switching from low‐ to medium‐, and from low‐ to high‐efficacy DMTs, before and after matching.

	Prior to matching			After matching		
	Patients switching from low‐ to medium‐efficacy DMTs	Patients switching from low‐ to high‐efficacy DMTs	SMD	Patients switching from low‐ to medium‐efficacy DMTs	Patients switching from low‐ to high‐efficacy DMTs	SMD
***n***	717	320		225	225	
**Age, years, mean (SD)**	44.6 (9.75)	42.0 (10.9)	***0.256***	42.4 (9.9)	42.2 (10.6)	0.020
**Gender, female, number (%)**	504 (70.3)	236 (73.8)	0.077	171 (76.0)	163 (72.4)	0.081
**EDSS, median [IQR]**	2.5 [2–3.5]	3.5 [2.5–4]	***0.627***	3.0 [2–4]	3.0 [2–4]	0.097
**Disease duration, years, mean (SD)**	15.9 (6.8)	11.7 (7.4)	0.088	12.1 (7.1)	11.9 (7.6)	0.046
**No of previous DMTs, mean (SD), median**	1.10 (0.32), 1	1.07 (0.30), 1	0.107	1.14 (0.37), 1	1.08 (0.31), 1	0.194
**DMT prior to escalation**			0.103			0.063
**Interferon beta‐1a**	401 (55.9)	173 (54.1)		131 (58.2)	124 (55.1)	
**Peginterferon beta‐1a**	1 (0.1)	1 (0.3)		1 (0.4)	1 (0.4)	
**Interferon beta‐1b**	194 (27.1)	99 (30.9)		60 (26.7)	65 (28.9)	
**Glatirameracetate**	121 (16.9)	47 (14.7)		33 (14.7)	35 (15.6)	
DMT after escalation*			***2.09***			***1.948***
**Fingolimod**	591 (82.4)	0 (0.0)		191 (84.9)	0 (0.0)	
**Dimethyl fumarate**	126 (17.6)	0 (0.0)		34 (15.1)	0 (0.0)	
**Ocrelizumab**	0 (0.0)	37 (11.6)		0 (0.0)	34 (15.1)	
**Natalizumab**	0 (0.0)	283 (88.4)		0 (0.0)	191(84.9)	
**Duration on prior DMT, years, mean (SD)**	3.6 (2.3)	2.5 (2.2)	***0.516***	2.9 (2.0)	2.8 (2.5)	0.072
**Relapse last year, yes (%)**	235 (32.8)	269 (84.1)	***1.219***	180 (80)	177 (78.7)	0.033
**Relapse last 2 years, yes (%)**	353 (49.2)	292 (91.2)	***1.035***	195 (86.7)	198 (88.0)	0.040
**No of relapses in the previous year, mean (SD)**	0.54 (0.95)	1.74 (1.37)	***0.908***	1.29 (1.24)	1.41 (1.26)	0.096
**All previous Relapses, mean (SD)**	2.03 (2.05)	2.73 (2.13)	***0.332***	2.56 (2.06)	2.57 (2.03)	0.009
**Time since last relapse, years, mean (SD)**	3.5 (3.7)	0.8 (1.8)	***0.908***	1.2 (2.4)	1.0 (2.0)	0.072

*Note*: A SMD of < 0.2 is considered a sign of adequate balance. SMDs > 0.2 are displayed in *bold and italic* numbers.

*DMT after escalation was not included in the matching procedure.

Abbreviations: DMT, disease modifying drug; EDSS, Expanded Disability Status Scale; SD, standard deviation; SMD, standardized mean difference.

### 3.2 Primary analysis

**FIGURE 3:**
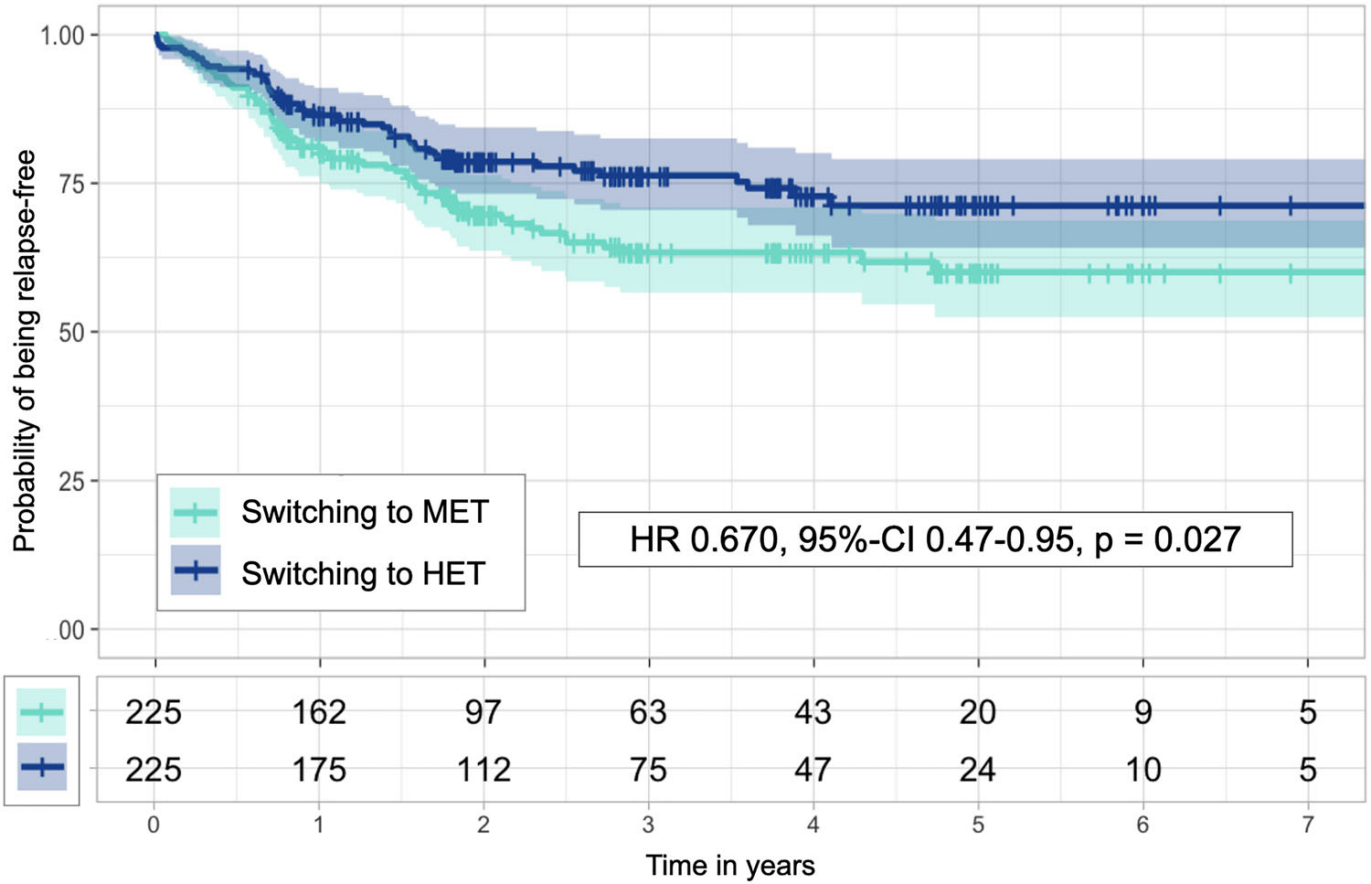
Kaplan–Meier curves (upper panel) and numbers at risk (lower panel) for patients escalating to MET (turquoise) and patients escalating to HET (dark blue), as well as results from Cox proportional hazard model. HR, hazard risk; 95% CI, 95% confidence interval.

The matching yielded 225 well‐balanced pairs (all matching variables SMDs < 0.2, Figure 2). The median follow‐up to pair‐wise censoring was 1.9 years (IQR 2.79), corresponding to 1141 person‐years. During follow‐up, 125 outcome events occurred, hereof 73 [58%] in patients switching to MET, and 52 [42%] in patients switching to HET. The assumption of proportionality was met. The hazard for relapses was lower in patients escalating to HET versus to MET (HR = 0.670, 95% CI 0.47–0.95, *p *= .027, Figure 3). Patients switching to HET had a lower ARR than switching to MET (RR = 0.70, 95% CI 0.50–0.99, *p *= .04). We found no evidence for a difference in cumulative hazards of 12‐month confirmed EDSS worsening (27 events, hereof 9 in patients switching to HET, and 18 in patients switching to MET; HR = 0.49, 95% CI 0.22–1.09, *p *= .07).

### 3.3 Secondary analyses

**FIGURE 4:**
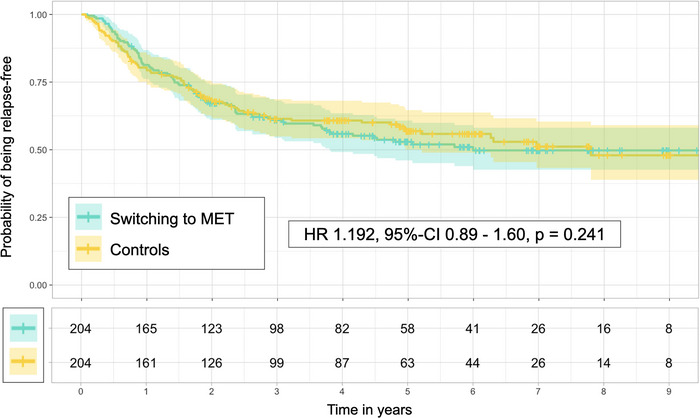
Kaplan–Meier curves (upper panel) and numbers at risk (lower panel) for patients switching to MET (turquoise) and controls (yellow), as well as results from Cox proportional hazard model. HR, hazard ratio; MET, medium‐efficacy treatment; 95% CI, 95% confidence interval.

We identified 720 patients who remained on interferon‐beta or glatiramer acetate during the entire follow‐up (i.e. “controls”). When considering each visit of these patients as potential matching timepoint (*n *= 2488), the matching with the 225 patients escalating to MET yielded 204 well‐balanced pairs (Supplementary Table 1; median [IQR] follow‐up 2.9 [4.4] years, 1497 patient‐years; 179 relapses, hereof 87 [48.6%] in patients escalating to MET, 92 [51.4%] in controls). The study found no evidence of a difference in the cumulative hazards of relapses (HR = 1.19, 95% CI 0.89–1.60, *p *= .2, Figure 4), or in the ARR (RR = 0.93, 95% CI 0.66–1.34, *p *= .7). The hazards for EDSS worsening were comparable (39 events, hereof 18 in patients switching to MET, 21 in controls; HR = 0.96, 95% CI 0.49–1.88, *p *= .9).

**FIGURE 5:**
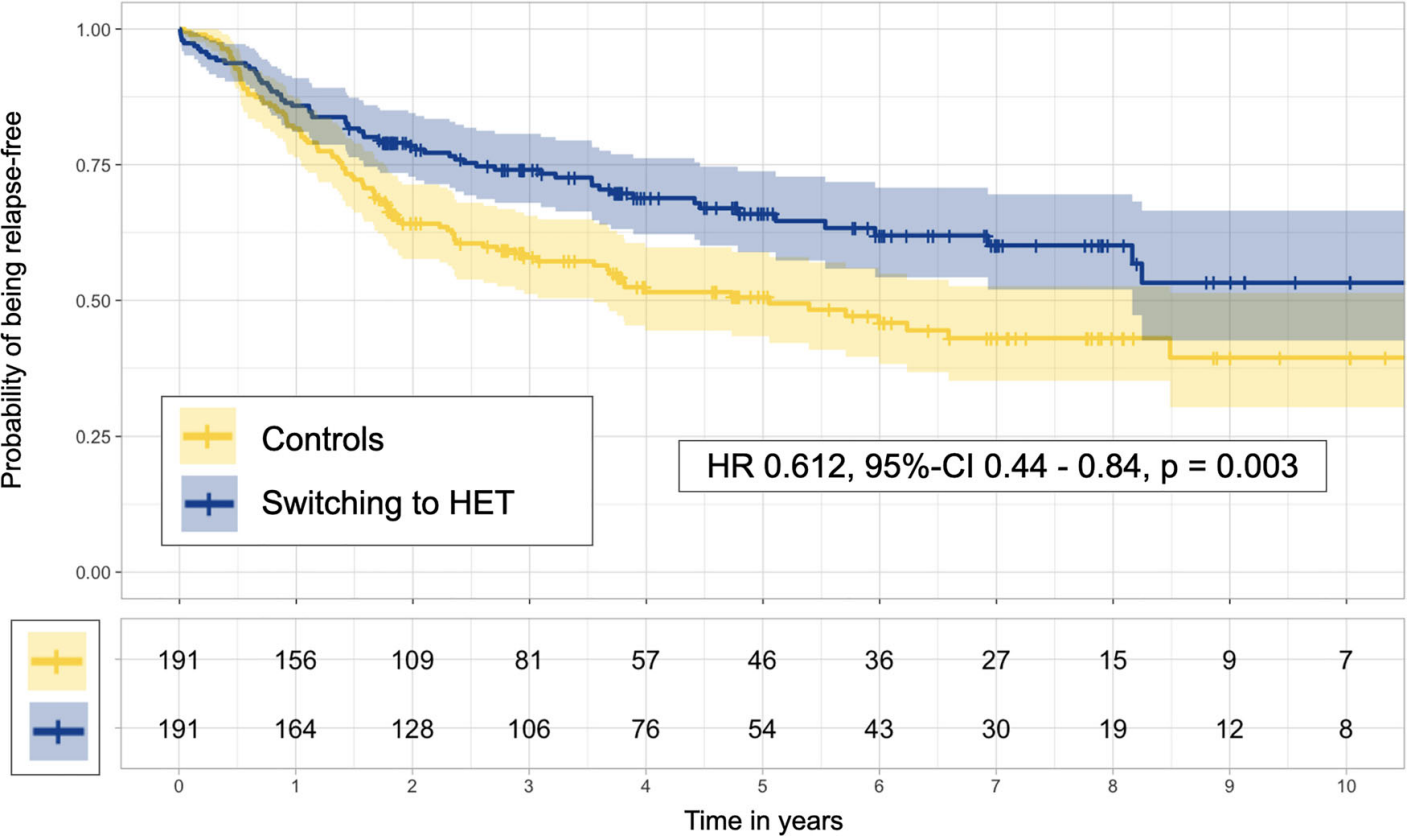
Kaplan–Meier curves (upper panel) and numbers at risk (lower panel) for patients escalating to HET (dark blue) and controls (yellow), as well as results from Cox proportional hazard model. HET, high‐efficacy treatment; HR, hazard ratio; 95% CI, 95% confidence interval.

Matching the 225 patients who escalated to HET to the 2488 potential matching timepoints of controls resulted in 191 well‐balanced pairs (Supplementary Table 2; median [IQR] follow‐up 3.0 [3.4] years, 1414 patient‐years; 158 relapses, hereof 64 [40.5%] in patients escalating to HET, 94 [59.5%] in controls). Patients switching to HET had a lower hazard of relapses (HR = 0.612, 95% CI 0.44–0.84, *p = *.003, Figure 5) and a lower ARR (RR = 0.48, 95% CI 0.32–0.73, *p *< .001). This analysis did not find any evidence of a differences in the EDSS worsening (35 events, hereof 13 in patients escalating to HET, 22 in controls, HR = 0.57, 95% CI 0.27–1.20, *p *= .1)

### 3.4 Sensitivity analysis

In patients *with* a relapse in the year before escalation (*n *= 504, hereof 235 in patients switching to MET, 269 in patients switching to HET; 183 matched pairs), the study was not sufficiently powered to identify differences in the hazards of relapses and ARRs between the two groups (HR = 0.78, 95% CI 0.55–1.10, *p *= .15; RR = 1.28, 95‐CI 0.89–1.86, *p *= .19). A similar observation was made in patients *without* a relapse (*n *= 533, hereof 482 switching to MET, 51 switching to HET; 46 matched pairs, HR = 0.73, 95% CI 0.28–1.90, *p *= .52; RR = 1.7, 95% CI 0.6–4.9, *p *= .31).

When following an “intention to treat” approach, the results of the primary time‐to‐event‐analysis were confirmed (median [IQR] follow‐up 2.5 [2.6] years, 1366 patient‐years; 164 events, hereof 92 in patients escalating to MET, 72 in patients switching to HET; HR = 0.72, 95% CI 0.53–0.98, *p *= .036), but the ARR was comparable (RR = 1.09, 95% CI 0.89–1.52, *p *= .58).

## 4 DISCUSSION

In our cohort of RRMS patients from Switzerland, we found that patients escalating from a low‐ to high‐efficacy therapy showed longer periods to disease activity than patients switching from low‐ to moderate‐efficacy therapy. While there was a clear benefit in patients who pursued an escalation to HET compared to nonswitching controls, we did not find evidence of a difference between patients who followed an escalation to MET compared to nonswitching controls. Taken together, our observation suggests that, once escalation from a low‐efficacy DMT is indicated, switching directly to a high‐efficacy treatment is superior to a stepwise escalation starting with a moderate‐efficacy treatment.

These results imply that patients initially treated with an escalation approach (i.e., first treatment with a low‐efficacy DMT) could benefit more from a transition to a high‐efficacy treatment than from a change to a moderate‐efficacy treatment, once a treatment escalation is needed or desired. While the finding of better disease control on high‐efficacy DMT is not surprising and is in line with a body of comparative real‐world evidence on related topics (Brown et al., 2019; Buron et al., 2020; Harding et al., 2019; He et al., 2020; Iaffaldano et al., 2021; Kalincik et al., 2015; Simonsen et al., 2021; Spelman et al., 2021), the absence of a discernible difference between switchers to MET and controls is somewhat unexpected, given that several comparative effectiveness (Boster et al., 2017; Group et al., 2018) studies consistently confirmed the higher efficacy of oral therapies in preventing relapses than baseline injectables. Furthermore, it is in contrast to a study that showed better outcomes in 148 patients switching from low‐efficacy DMTs to fingolimod than 379 patients switching to another low‐efficacy DMT following on‐treatment clinical disease activity (He et al., 2015). In our study, it is plausible that patients on low‐efficacy treatment who later required escalation represent a specific subgroup of patients with a particularly active disease, as evidenced by the relatively high proportion of patients with a relapse prior to escalation (80% in the moderate escalation group). To counteract this potential bias, we employed a matching approach in which every visit of the controls could serve as potential matching time point and as baseline. It is possible that residual indication bias persisted, potentially causing a situation where the oral DMT was able to attenuate the disease activity toward the level observed in the controls. Consequently, the beneficial effect of oral DMTs compared to injectables might not have reached statistical significance within the relatively short median follow‐up of 2.9 years. This is in contrast to the comparison between controls and patients escalating directly to HET, where the favorable effect of the high‐efficacy treatment was clearly evident. The assumption of residual indication bias, however, is opposed by our well‐balanced matching for variables describing preswitch disease activity, such as presence of relapses in the past one (patients escalating to MET vs. controls: 77.9 vs. 81.4%) and two years (85.3 vs. 86.3%), time since last relapse (15.4 vs. 14.6 months), ARR in the year prior to escalation (1.17 vs. 1.03), and total number of relapses (2.56 vs. 2.48; SMD < 0.2 for all variables). Even though these numbers indicate a good balance, residual indication bias may be introduced by unknown confounding variables. An example is relapse severity or reason for treatment escalation, which are not routinely and explicitly reported in the SVK. Hence, it remains possible that patients experiencing less severe relapses (e.g., affecting only the sensory system) continued with low‐efficacy treatment, while those with more severe relapses consequently switched to higher‐efficacy treatment.

A previous study compared clinical outcomes of patients switching from interferons to higher‐efficacy treatment, albeit in a slightly different context: In 2014, prior to the approval of many DMTs that are available today (particularly before ocrelizumab and dimethyl fumarate), and before the emerging trend of early aggressive treatment, Kalincik et al. (2015) compared patients with disease breakthrough on interferon beta or glatiramer acetate, who switched to either natalizumab or fingolimod. They observed reduced ARR and extended times to next relapse in patients escalating directly to high‐effective treatment, closely aligning with the results of our primary analysis. Our study further builds on these findings by broadening the scope and including patients regardless of their preswitch disease activity, thereby encompassing switches due to adverse events or patient preference, amongst others. Additionally, in contrast to the aforementioned study, we deliberately chose to include multiple DMTs per intensity group, in order to rather provide a comparison of treatment strategies, as opposed to a comparison of single DMT compounds and their effectiveness.

A number of observational cohort studies have assessed treatment strategies in treatment‐naive or general MS populations (Brown et al., 2019; Buron et al., 2020; Harding et al., 2019; He et al., 2020; Iaffaldano et al., 2021; Simonsen et al., 2021). An extensive analysis of our study's results in context of these studies is given in the Supplementary Text 2. In summary, this body of evidence is in support of the *early‐intensive* approach, whether if it is when starting DMT in treatment‐naive patients, or when escalating from baseline injectables, as further demonstrated by our study. However, caution should be exercised before drawing rigid conclusions based on these studies. Variability in study design, statistical methodologies, diverse cohorts, and discrepancies in DMT intensity classifications across studies introduce complexities in interpretations and direct comparisons.

The main limitations of our study arise from its nonrandomized observational study design and the concomitant susceptibility to systematic biases, notably indication bias (Signorello et al., 2002), attrition bias (Lewin et al., 2018), immortal time bias (Suissa, 2007), informed presence bias (Goldstein et al., 2016), ascertainment bias (Greene et al., 2021), and Will‐Rogers phenomenon (Sormani et al., 2008). A detailed discussion of these biases in context of our study along with our measures taken against them is given in the Supplementary Text 3. Another limitation is the lack of additional biomarkers: Conventional or advanced MRI images and fluid biomarkers such as neurofilament light chains provide additional information on subclinical disease activity and could drive strategic decision‐making in daily clinical practice. Likewise, the dataset lacks information on the reasoning behind the escalation, as determined by the treating neurologist, as well as on potential adverse events. Information on these parameters would give a more comprehensive context of the escalation. In a sensitivity analysis, we restricted the population to patients with a relapse in the year prior to escalation to stratify for this escalation reason, but this analysis yielded underpowered results. Hence, future studies should place greater emphasis on factors influencing escalation considerations such as adverse events and DMT tolerability, as these aspects are considered particularly relevant by proponents of the escalation approach but are unfortunately not routinely collected in our cohort. Last but not least, it is essential to acknowledge that the present study may not entirely reflect the complexity of treatment selection, as treating physicians may make their decision based on a complex interplay of multiple factors, including considerations that are not fully represented in this study, such as comorbidities and patient preferences, among others.

## 5 CONCLUSION

In summary, our study on real‐world data from a cohort of RRMS patients in Switzerland provides evidence in favor of a direct treatment escalation to high‐efficacy DMTs, in patients who are initially on low‐efficacy DMT, but require a change of treatment. In our cohort, the effect of an escalation to medium‐efficacy DMT was relatively limited compared to continued application of low‐efficacy drugs. Therefore, it advocates for the adoption of an early therapy with high‐efficacy DMT, endorsing a shift in the treatment strategy for patients who initially followed an escalation approach by starting with low‐efficacy DMTs.

## AUTHOR CONTRIBUTIONS

**Jannis Müller**: Conceptualization; methodology; formal analysis; visualization; writing—original draft; writing—review and editing. **Izanne Roos**: Conceptualization; methodology; formal analysis; supervision; writing—review and editing. **Tomas Kalincik**: Conceptualization; methodology; formal analysis; supervision; writing—review and editing. **Johannes Lorscheider**: Conceptualization; methodology; writing—review and editing. **Edoardo Galli**: Methodology; writing—review and editing. **Pascal Benkert**: Conceptualization; methodology; writing—review and editing. **Sabine Schädelin**: Conceptualization; methodology; writing—review and editing. **Sifat Sharmin**: Conceptualization; methodology; writing—review and editing. **Maximilian Einsiedler**: Conceptualization; writing—review and editing. **Peter Hänni**: Data curation; resources; writing—review and editing; project administration. **Jürg Schmid**: Data curation; resources; writing—review and editing; project administration. **Jens Kuhle**: Conceptualization; methodology; writing—review and editing. **Tobias Derfuss**: Conceptualization; writing—review and editing; methodology. **Cristina Granziera**: Conceptualization; methodology; writing—review and editing. **Tjalf Ziemssen**: Conceptualization; methodology; writing—review and editing. **Timo Siepmann**: Conceptualization; methodology; supervision; formal analysis; writing—review and editing; visualization. **Özgür Yaldizli**: Conceptualization; methodology; visualization; writing—review and editing; formal analysis; supervision.

## FUNDING

There was no project‐specific funding for this work.

## CONFLICT OF INTEREST STATEMENT

*Jannis Müller* has nothing to disclose about this work. He has received financial support by the Swiss National Science Foundation (grant No. P500PM_214230). *Izanne Roos* has reported receiving grants from MSIF, MS Australia, and the Trish Multiple Sclerosis Research Foundation and served on scientific advisory boards, received conference travel support and/or speaker honoraria from Roche, Novartis, Merck, and Biogen outside the submitted work. *Tomas Kalincik* served on scientific advisory boards for MS International Federation and World Health Organisation, BMS, Roche, Janssen, Sanofi Genzyme, Novartis, Merck and Biogen, steering committee for Brain Atrophy Initiative by Sanofi Genzyme, received conference travel support and/or speaker honoraria from WebMD Global, Eisai, Novartis, Biogen, Roche, Sanofi Genzyme, Teva, BioCSL, and Merck, and received research or educational event support from Biogen, Novartis, Genzyme, Roche, Celgene, and Merck. *Johannes Lorscheider* received research support from Innosuisse‐Swiss Innovation Agency, Biogen and Novartis and served on advisory boards for Roche and Teva. *Edoardo Galli* declares that there is no conflict of interest. *Pascal Benkert* declares that there is no conflict of interest. *Sabine Schädelin* declares that there is no conflict of interest. *Sifat Sharmin* declares that there is no conflict of interest. *Maximilian Einsiedler* declares that there is no conflict of interest. *Peter Hänni* is an employee of the Swiss Federation for Common Tasks of Health Insurances *Jürg Schmid* is an employee of the Swiss Federation for Common Tasks of Health Insurances. *Jens Kuhle* received speaker fees, research support, travel support, and/or served on advisory boards by Swiss MS Society, Swiss National Research Foundation (320030_189140/1), University of Basel, Progressive MS Alliance, Alnylam, Bayer, Biogen, Bristol Myers Squibb, Celgene, Immunic, Merck, Neurogenesis, Novartis, Octave Bioscience, Quanterix, Roche, Sanofi, Stata DX. *Tobias Derfuss* received speaker fees, research support, travel support, and/or served on Advisory Boards or Steering Committees of Alexion, Novartis, Merck, Biogen, GeNeuro, MedDay, Roche, and Sanofi Genzyme. *Cristina Granziera* has received the following fees which were used exclusively for research support: (i) advisory board and consultancy fees from Actelion, Genzyme‐Sanofi, Novartis, GeNeuro, and Roche; (ii) speaker fees from Genzyme‐Sanofi, Novartis, GeNeuro and Roche; (iii) research support from Siemens, GeNeuro, Roche. Cristina Granziera is supported by the Swiss National Science Foundation (SNSF) grant PP00P3_176984, the Stiftung zur Förderung der gastroenterologischen und allgemeinen klinischen Forschung, and the EUROSTAR E!113682 HORIZON2020. *Tjalf Ziemssen* received personal compensation from Biogen, Bayer, Celgene, Novartis, Roche, Sanofi, Teva for the consulting services. Ziemssen received additional financial support for the research activities from Bayer, Biogen, Novartis, Teva, and Sanofi. *Timo Siepmann* received grants from the German Federal Ministry of Health, Kurt Goldstein Institut, German Parkinson Association that were not related to this study. Dr. Siepmann received royalties from Thieme as well as from Astrazeneca for consulting and from Dresden International University for serving as program director and lecturer of the Master's Program in Clinical Research. *Özgür Yaldizli* received grants from Swiss National Science Foundation, ECTRIMS/MAGNIMS, University of Basel, Pro Patient Stiftung University Hospital Basel, Free Academy Basel, Swiss Multiple Sclerosis Society and advisory board, lecture and consultancy fees from Roche, Sanofi Genzyme, Allmirall, Biogen, and Novartis.

### PEER REVIEW

The peer review history for this article is available at https://publons.com/publon/10.1002/brb3.3498.

## Supporting information

Supplementary Information

## Data Availability

The data are accessible on reasonable request at the Department of Clinical Research, University of Basel via the corresponding author.
