# Supplementary material for: Escalating to medium‐ versus high‐efficacy disease modifying therapy after low‐efficacy treatment in relapsing remitting multiple sclerosis
Source: Brain Behav. 2024 Apr 30;14(5):e3498. doi: 10.1002/brb3.3498 (PMC11061202; doi:10.1002/brb3.3498)
Supplement: Supplementary file 1 — Supplementary Information [file BRB3-14-e3498-s001.docx]

**Supplementary Material**

**Supplementary Table 1**

|  | **Prior to matching** | | | **After matching** | | |
| --- | --- | --- | --- | --- | --- | --- |
|  | **Control Visits*** | **Patients switching from low- to medium-efficacy DMTs** | **SMD** | **Controls** | **Patients switching from low- to medium-efficacy DMTs** | **SMD** |
| **n** | 2488 | 225 |  | 204 | 204 |  |
| **Age, years, mean (SD)** | 49.2 (10.4) | 42.4 (9.9) | ***0.672*** | 43.1 (9.4) | 43.1 (9.9) | 0.002 |
| **Gender, female, number (%)** | 1829 (73.5) | 171 (76.0) | 0.057 | 153 (75.0) | 157 (77.0) | 0.046 |
| **EDSS, mean (SD)** | 2.55 (1.20) | 3.0 [2-4] | ***0.385*** | 2.97 (1.36) | 2.96 (1.16) | 0.006 |
| **Disease duration, years, mean (SD)** | 14.53 (8.9) | 12.1 (7.1) | ***0.300*** | 12.1 (8.0) | 12.3 (7.2) | 0.030 |
| **No of previous DMTs, mean (SD)§** | 0 (0.0) | 1.14 (0.37), 1 | ***4.311*** | 0 (0.0) | 1.13 (0.36) | ***4.481*** |
| **DMT prior to escalation** |  |  | 0.130 |  |  | 0.116 |
| **Interferon beta-1a** | 1385 (55.6) | 131 (58.2) |  | 117 (57.4) | 119 (58.3) |  |
| **Peginterferon beta-1a** | 1 (0.1) | 1 ( 0.4) |  | 0 (0.0) | 0 (0.0) |  |
| **Interferon beta-1b** | 645 (25.9) | 60 (26.7) |  | 62 (30.4) | 55 (27.0) |  |
| **Glatirameracetate** | 457 (18.4) | 33 (14.7) |  | 25 (12.3) | 30 (14.7) |  |
| **DMT after escalation** |  |  | ***14.152*** |  |  | ***1.466*** |
| **Fingolimod** | 0 (0.0) | 191 (84.9) |  | 0 (0.0) | 172 (84.3) |  |
| **Dimethyl fumarate** | 0 (0.0) | 34 (15.1) |  | 0 (0.0) | 32 (15.7) |  |
| **Interferon beta-1a** | 1385 (55.6) | 0 ( 0.0) |  | 117 (57.4) | 0 (0.0) |  |
| **Peginterferon beta-1a** | 1 (0.1) | 0 ( 0.0) |  | 0 (0.0) | 0 (0.0) |  |
| **Interferon beta-1b** | 645 (25.9) | 0 ( 0.0) |  | 62 (30.4) | 0 (0.0) |  |
| **Glatirameracetate** | 457 (18.4) | 0 ( 0.0) |  | 25 (12.3) | 0 (0.0) |  |
| **Duration on prior DMT, years, mean (SD)** | 2.9 (2.1) | 2.9 (2.0) | 0.002 | 2.8 (2.1) | 2.9 (1.9) | 0.061 |
| **Relapse last year, yes (%)** | 325 (13.1) | 180 (80) | ***1.810*** | 166 (81.4) | 159 (77.9) | 0.085 |
| **Relapse last 2 years, yes (%)** | 714 (28.7) | 195 (86.7) | ***1.449*** | 176 (86.3) | 174 (85.3) | 0.028 |
| **No of relapses in the previous year, mean (SD)** | 0.18 (0.53) | 1.29 (1.24) | ***1.169*** | 1.03 (1.13) | 1.17 (1.14) | 0.121 |
| **All previous Relapses, mean (SD)** | 1.36 (1.93) | 2.56 (2.06) | ***0.599*** | 2.48 (2.63) | 2.56 (2.06) | 0.037 |
| **Time since last relapse, years, mean (SD)** | 4.6 (3.8) | 1.2 (2.4) | ***1.179*** | 1.2 (2.2) | 1.3 (2.5) | 0.027 |
| **Supplementary Table 1: Baseline characteristics before and after matching of patients escalating to medium efficacy DMT and controls, who remained on baseline injectables.** *Note that the values in the pre-matching controls are summarizing all visits and should be therefore interpreted with caution. §Matching was deliberately performed without the „No of previous DMTs“ because this variable has defining character of the groups. **Abbreviations:** DMT disease modifying drug; EDSS expanded disability status scale; SD standard deviation; SMD standardized mean difference. | | | | | | |

**Supplementary Table 2**

|  | **Prior to matching** | | | **After matching** | | |
| --- | --- | --- | --- | --- | --- | --- |
|  | **Control Visits*** | **Patients switching from low- to high-efficacy DMTs** | **SMD** | **Controls** | **Patients switching from low- to high-efficacy DMTs** | **SMD** |
| **n** | 2488 | 225 |  | 191 | 191 |  |
| **Age, years, mean (SD)** | 49.2 (10.4) | 42.2 (10.6) | ***0.668*** | 42.5 (9.2) | 42.9 (10.7) | 0.043 |
| **Gender, female, number (%)** | 1829 (73.5) | 163 (72.4) | 0.024 | 145 (75.9) | 141 (73.8) | 0.048 |
| **EDSS, mean (SD)** | 2.55 (1.20) | 3.0 [2-4] | ***0.479*** | 2.98 (1.41) | 3.04 (1.21) | 0.044 |
| **Disease duration, years, mean (SD)** | 14.53 (8.9) | 11.9 (7.6) | ***0.324*** | 12.3 (8.0) | 12.3 (7.7) | 0.007 |
| **No of previous DMTs, mean (SD)** | 0 (0.0) | 1.08 (0.31), 1 | ***4.885*** | 0 (0.0) | 1.08 (0.33) | ***4.648*** |
| **DMT prior to escalation** |  |  | 0.123 |  |  | 0.105 |
| **Interferon beta-1a** | 1385 (55.6) | 124 (55.1) |  | 98 (51.3) | 105 (55.0) |  |
| **Peginterferon beta-1a** | 1 (0.1) | 1 ( 0.4) |  | 0 (0.0) | 0 (0.0) |  |
| **Interferon beta-1b** | 645 (25.9) | 65 (28.9) |  | 61 (31.9) | 54 (28.3) |  |
| **Glatiramer acetate** | 457 (18.4) | 35 (15.6) |  | 32 (16.8) | 32 (16.8) |  |
| **DMT after escalation** |  |  | ***14.152*** |  |  | ***1.405*** |
| **Natalizumab** | 0 (0.0) | 34 (15.1) |  | 0 (0.0) | 159 (83.2) |  |
| **Ocrelizumab** | 0 (0.0) | 191(84.9) |  | 0 (0.0) | 32 (16.8) |  |
| **Interferon beta-1a** | 1385 (55.6) | 34 (15.1) |  | 98 (51.3) | 0 (0.0) |  |
| **Peginterferon beta-1a** | 1 (0.1) | 0 (0.0) |  | 0 (0.0) | 0 (0.0) |  |
| **Interferon beta-1b** | 645 (25.9) | 0 (0.0) |  | 61 (31.9) | 0 (0.0) |  |
| **Glatirameracetate** | 457 (18.4) | 0 (0.0) |  | 32 (16.8) | 0 (0.0) |  |
| **Duration on prior DMT, years, mean (SD)** | 2.9 (2.1) | 2.8 (2.5) | 0.068 | 2.6 (1.9) | 2.7 (2.3) | 0.047 |
| **Relapse last year, yes (%)** | 325 (13.1) | 177 (78.7) | ***1.749*** | 150 (78.5) | 143 (74.9) | 0.087 |
| **Relapse last 2 years, yes (%)** | 714 (28.7) | 198( 88.0) | ***1.506*** | 163 (85.3) | 164 (85.9) | 0.015 |
| **No of relapses in the previous year, mean (SD)** | 0.18 (0.53) | 1.41 (1.26) | ***1.273*** | 1.08 (1.19) | 1.18 (1.10) | 0.087 |
| **All previous Relapses, mean (SD)** | 1.36 (1.93) | 2.57 (2.03) | ***0.612*** | 2.65 (2.53) | 2.60 (2.12) | 0.022 |
| **Time since last relapse, years, mean (SD)** | 4.6 (3.8) | 1.0 (2.0) | ***1.186*** | 1.2 (2.1) | 1.2 (2.2) | 0.007 |
| **Supplementary Table 2: Baseline characteristics before and after matching of aggressive escalators and controls, who remained on baseline injectables.** *Note that the values in the pre-matching controls are summarizing all visits and should be therefore interpreted with caution. Matching was deliberately performed without the „No of previous DMTs“ because this variable has defining character of the groups. **Abbreviations:** DMT disease modifying drug; EDSS expanded disability status scale; SD standard deviation; SMD standardized mean difference. | | | | | | |

**Supplementary Text 1: Steps undertaken to assure data quality**

- Duplicates records were removed
- Birth date was checked and patients with an implausible date of birth (e.g. 01.01.1900) or with a missing date of birth were excluded
- A plausible sequence of MS disease course was assured (e.g. clinically isolated syndrome, followed by relapsing remitting MS, followed by secondary progressive disease course)
- Date of first clinical presentation was checked and patients with an implausible or missing date of first clinical presentation were excluded
- Patients with a reported age of <0 or >100 years at MS onset were excluded
- Relapses that were reported prior to the date of „first symptoms“ were deleted
- Data entries with implausible number of relapses in the previous year (e.g. >12/year) were excluded
- Visits prior to 01.01.2007 were excluded (before, none of the escalating drugs assessed in this study were licensed in Switzerland).
- Data entries with missing date, or with the recorded date before the clinical onset of MS were removed
- Data entries with erroneous EDSS (e.g. EDSS <0 or EDSS >10) were excluded

**Supplementary Text 2: Extended discussion on the study results in context of previous studies**

To the best of our knowledge, only one study compared clinical outcomes of patients switching from interferons to higher-efficacy treatment, albeit in a slightly different context: In 2014, prior to the approval of many DMTs that are available today (particularly before ocrelizumab and dimethyl fumarate), and before the emerging trend of early aggressive treatment, Kalincik *et al.*^1^ conducted a study on patients with breakthrough disease activity on interferon beta or glatiramer acetate.^1^ They compared patients who switched to natalizumab with patients that transitioned to fingolimod. After a median follow-up of 12 months, they observed superior outcomes in the first group, encompassing reduced annualized relapse rates and extended times to next relapse. These results align closely with the results of our primary analysis. However, our study builds on these findings by broadening the scope and including patients regardless of their pre-switch disease activity, thereby encompassing switches due to adverse events or patient preference, amongst others. Additionally, in contrast to the previous study, we deliberately chose to include multiple DMTs per intensity group, in order to rather provide a comparison of treatment strategies, as opposed to a comparison of single DMT compounds and their effectiveness.

There is a number of observational cohort studies evaluating treatment strategies in treatment-naive MS patients or in the general MS population, that allow us to put our study in context: An analysis by Brown *et al.^2^* studied data of 1555 treatment-naive RRMS patients from the international multicenter MSBase cohort and the Cardiff cohort. It showed that patients initially treated with fingolimod, natalizumab or alemtuzumab had a lower risk of converting to SPMS than patients initially treated with interferon beta or glatiramer acetate.^2^ This finding underscored the high relevance of an early intensive treatment not only with regard to short-term, but also to long-term prognosis. Another population based analysis of 592 individuals from the United Kingdom demonstrated that patients who received high-efficacy DMTs (natalizumab or alemtuzumab) as first-line therapy showed smaller increases in their EDSS score after 5 years, compared to those who started with moderate-efficacy DMTs (interferons, glatiramer acetate, dimethyl fumarate, fingolimod or teriflunomide).^3^ Similar outcomes were found in a population-based study from Denmark,^4^ in which 194 patients starting with high-efficacy treatment (natalizumab, fingolimod, alemtuzumab) had a lower risk of EDSS worsening compared to 194 propensity score-based matched patients with low/medium efficacy treatment (interferon beta, glatiramer acetate, teriflunomide) after a follow-up of 4 years. Similarly, in a Norwegian cohort containing 594 treatment-naive patients, those who initiated treatment with high-efficacy DMTs (n = 103) were more likely to achieve absence of disease activity (including relapses, progression or MRI activity) at year 1 and 2 than the 491 matched patients initiating moderate-efficacy drugs.^5^ In summary, these studies provide evidence in favor of a strategy that initiates high-efficacy DMTs as first therapy in treatment-naive patients. Adding to this body of evidence, our study expanded the target population to patients who had already started treatment but needed DMT escalation, a scenario which is very frequently encountered in daily clinical practice.

The two following studies used a slightly different approach to provide evidence on treatment strategies: Instead of investigating treatment-naive patients and comparing the initiation of low- efficacy vs. high-efficacy treatment, they compared the start of high-efficacy DMTs early in the disease course vs. late in the disease course. The first study was conducted within the MSBase cohort, in which He *et al*. compared patients who started high-efficacy treatment either 0 - 2 years („early initiation“, n = 277) or 4 - 6 years („late initiation“, n = 267) after clinical disease onset. They found that an early initiation was associated with less disability after 6-10 years.^6^ A similar comparison of early vs. delayed (i.e., > 1 year on low-efficacy treatment before switching to high-efficacy treatment) treatment in 363 propensity score-based matched pairs from the Italian MS cohort showed that early intensive treatment was associated with a lower probability of disability progression, as measured by the mean annual EDSS change, up to 10 years after baseline.^7^ These findings provide further support for the existence of the previously discussed concept of a „window of opportunity“ in MS, in which an early intervention has the potential to modulate the MS disease course, achieving superior long-term outcomes.^8-11^

One recent study on treatment strategies deserves particular mentioning: Spelman et al.^12^ employed a unique approach, contrasting treatment outcomes in Sweden and Denmark, both of which adopt distinct therapeutic strategies: In Denmark, MS care workers tend to follow an *escalation* strategy, while in Sweden, MS professionals lean toward a *early-intensive* approach, as evidenced by a larger proportion of patients starting treatment with interferon or teriflunomide in the Danish MS registry, versus a larger share of patients initiating therapy with dimethyl fumarate, rituximab, or natalizumab in the Swedish MS registry. The study showed that patients in Sweden (n = 2700) had considerably longer intervals to subsequent relapses and confirmed disability worsening compared to their Danish counterparts (n = 2161) over a mean follow-up of 4.1 years, overall favoring a *early-intensive* approach.^12^

**Supplementary Text 3: Extended discussion on potential bias affecting the internal and external validity of the study**

Although the findings of our study align with the broader narrative of previous studies, it is essential to recognize that data from observational studies demand a thorough evaluation for a number of systematical biases that may compromise the internal validity of the study. In the following section, we aim to discuss the most important of these biases in detail and provide context for their relevance in our study.

As shortly indicated above, *indication bias* is one of the most crucial challenges when analyzing observational data.^13,14^ It arises from non-random treatment exposure, where - for instance in our study - patients who „need“ a higher efficacy treatment according to the treating physician (e.g. to address a more aggressive disease) are more likely to receive an escalation to HET. This scenario may result in an overrepresentation of patients with an aggressive disease course in the group of patients switching to HET. In order to counteract this bias, we applied propensity score-based matching, encompassing a range of variables that capture disease activity in the years before treatment escalation (i.e., relapse within the previous year, relapses within the previous 2 years, time since last relapse and total number of relapses since disease onset). Statistical and visual assessments of the matching procedures confirmed that the matching was able to significantly increase the balance of the known confounding variables between the matched groups. Still, residual indication bias might be caused by unknown confounding factors or known confounders that are not covered in the matching procedure (e.g. relapse severeness, as mentioned above). However, our finding of superior outcomes in patients escalating to HET runs in the opposite direction of this bias, suggesting that the likelihood of indication bias is low in the primary analysis, or, that our results are strong enough to effectively overcome this bias.

Another critical systematic bias to consider in time-to-event analyses is *attrition bias*.^15-17^ It arises when there is systematically different loss to follow-up between the two groups, particularly if the characteristics of patients who drop out differ from those who continue to participate. Such attrition bias could change the characteristics of the groups independent of the treatment exposure, for instance, due to disease-unrelated loss of follow-up. To address this bias, we performed pair-wise censoring at the shorter time-interval of the matched pair. While this measure resulted in some loss of follow-up time and reduction in the number of detected outcome events, it mitigated the likelihood of attrition bias to some degree. Also, in addition to our primary „per protocol“-analysis, where outcomes were solely considered while the patient was on the first DMT after escalation, we also conducted a sensitivity analysis using an „intention to treat“-approach. This approach involved assessing future outcome events regardless of whether the patient was still under the treatment of consideration, therefore mitigating attrition bias. Notably, this analysis confirmed the findings of the primary analysis.

*Immortal time bias* is another systematic bias frequently encountered in cohort studies. It is caused when participants of one group cannot experience the outcome of interest because they have not yet received the intervention or treatment - even though they have been already included to the study. From a statistical time point of view, they are therefore effectively „immortal" during that time period.^18,19^ To address this concern, we aligned the start of follow-up with the date of the first reimbursement for the escalating drug, as opposed to synchronizing it with the last reimbursement of the previous DMT. This approach effectively aligned the treatment group assignment and the recorded start of follow-up. However, it is important to note that the date of the reimbursement is not necessarily the same as the date of the actual drug administration, theoretically leaving room for potential immortal time bias in both groups separately: Moderate efficacy treatments such as dimethyl fumarate or fingolimod require screening tests such as blood tests, cardiac, ophthalmologic and sometimes dermatologic assessment, potentially delaying the first drug administration even after reimbursement. Conversely, highly-efficacy treatments such as ocrelizumab or natalizumab are administered intravenously and may necessitate more logistic organization at treatment centers. This, too, could lead to delays in the first drug administration. Although there is no statistical method to fully control this bias, in order to overcome the time-dependency of our primary outcome, we additionally compared the annualized relapse rate after treatment escalation between the groups, which encompasses all recorded events after group allocation and is therefore less reliant on the precise date of the first administration. In our cohort, this rate was calculated as all relapses during follow-up until censoring, divided by the total number of follow-up, making it less susceptible to immortal time bias.

Last but not least, it is essential to acknowledge a potential bias deriving from the exclusive availability of treatment escalation options in patients who escalated to MET. Our primary analysis following a "per-protocol" approach, involved censoring at the point of treatment change. This approach may inadvertently loose events that genuinely pertain to the period under the moderate DMT. However, notably, the presence of this bias would diminish the group differences. Also, our sensitivity analysis following an „intention-to-treat" approach was less susceptible to this bias and was still able to corroborate our initial findings.

Besides these methodological limitations that may compromise the internal validity of the findings, several additional limitations should be considered that potentially reduce the external validity of our study. Most of them are closely related to the observational nature of our data: Selection bias, such as informed presence bias^20^ and ascertainment bias^21,22^ describe systematic differences in the identification and representation of individuals in a study population (in this case, the SVK database) compared to the target population. It is important to note that the SVK only covers 67% of the Swiss population. While it is impossible to entirely rule out selection bias in this setting, several factors make its presence less likely: First, inclusion to the SVK is determined by the patients choice of insurance company, and is not principally influenced by individual patient or disease characteristics. Second, coverage of MS care (including access to all available DMTs in Switzerland) is equally covered by all insurance companies. Third, all insurance providers offer both insurance models available in Switzerland (i.e. general and private insurance). Given these basic similarities, it is unlikely that the choice of insurance is systematically linked to specific patient attributes.

In comparison to other Swiss population based MS cohort studies, the SVK contains more patients on baseline injectables and less secondary progressing patients, but was otherwise comparable to other observational MS cohort studies from Switzerland.^23^ The differences in baseline characteristics between the SVK and other Swiss MS cohorts can be attributed to several factors: (I) Inclusion criteria. The SVK only includes patients with at least one DMT reimbursement form, whereas other cohorts include patients irrespective of their treatment status. Hence, it is not surprising that the SVK contains different shares of DMTs than other cohorts that also include untreated patients. Similarly, the limited treatment options for progressive MS forms may explain the lower proportion of these patients in the SVK, compared to cohorts that include patients irrespective of their disease phenotype. (II) Recruitment timing: The SVK initiated patient inclusion in 1995, which may result in a greater amount of data on „older“ DMTs such as injectables, compared to other cohorts. To mitigate this issue, we have restricted our analysis to data collected after 2007, but this historical difference may contribute to the disparities observed when cross-sectionally comparing entire Swiss MS cohorts. (III) Cohort Setting and Data Collection: While data of the SVK is sourced from board-certified neurologist in context of the routine reimbursement process, other Swiss cohorts, such as the Swiss MS Cohort are hosted by a consortium of all Swiss University Hospitals and two tertiary care centers across Switzerland, potentially leading to a selection of more severely affected patients in these cohorts. In multiple sclerosis, clinical definitions and classifications, e.g. for describing the clinical disease course (i.e., CIS vs. RRMS vs. SPMS, etc.), have undergone several changes over the past decades.^24-26^ This phenomenon was termed „Will-Rogers phenomenon“ and has been discussed as potential source of bias in longitudinal MS cohort studies.^27^ In addition to these shifts of disease classification, newly approved DMTs, changes of clinical practice and treatment recommendations over the years may potentially introduce bias into our longitudinal samples, in sense that the same patient might not have been classified and treated in the same manner decades ago, as he would be today. In order to mitigate the propensity for such bias to influence our results, we excluded data from before May 1, 2007. We chose this date because from then on, the first of the escalating DMTs assessed in this study (natalizumab) was approved for RRMS in Switzerland.

Last but not least, observational real-world cohorts have been criticized for their inherent limitations of quality, granularity and data precision, particularly when data is reported by treating physicians. In our study, we took extensive measures to ensure the robustness of our data: An independent data manager of the SVK performed rigorous assessments to verify data plausibility and completeness, prior to entering the data into the database. Additionally, we defined strict inclusion criteria requiring a minimal level of data completeness, and implemented extensive steps prior to the statistical analysis, in order to enhance the data quality (detailed steps are described in the Supplementary Text 1). Although this data cleaning resulted in a notable reduction in the number of included patients, it was instrumental in maintaining a reliable dataset and mitigating concerns related to data precision and granularity. Additionally, it is worth noting that a bias deriving from random reporting imprecision would create an environment that is prone to type II errors (false negatives), and to a lesser extent, type I errors (false positives). In other words, a negative finding (i.e., acceptance of the null hypothesis) may be attributed to data imprecision, because the inexact dataset fails to accurately reflect a difference that is genuinely present in the target population. Conversely, under the assumption that the data imprecision is completely random, a positive finding (i.e., rejection of the null hypothesis) as seen in our primary analysis, is less likely to be the false-positive result caused by a randomly imprecise dataset.

References of the Supplementary Material

1. Kalincik T, Horakova D, Spelman T, et al. Switch to natalizumab versus fingolimod in active relapsing-remitting multiple sclerosis. *Ann Neurol* 2015; **77**(3): 425-35.

2. Brown JWL, Coles A, Horakova D, et al. Association of Initial Disease-Modifying Therapy With Later Conversion to Secondary Progressive Multiple Sclerosis. *JAMA* 2019; **321**(2): 175-87.

3. Harding K, Williams O, Willis M, et al. Clinical Outcomes of Escalation vs Early Intensive Disease-Modifying Therapy in Patients With Multiple Sclerosis. *JAMA Neurol* 2019; **76**(5): 536-41.

4. Buron MD, Chalmer TA, Sellebjerg F, et al. Initial high-efficacy disease-modifying therapy in multiple sclerosis: A nationwide cohort study. *Neurology* 2020; **95**(8): e1041-e51.

5. Simonsen CS, Flemmen HO, Broch L, et al. Early High Efficacy Treatment in Multiple Sclerosis Is the Best Predictor of Future Disease Activity Over 1 and 2 Years in a Norwegian Population-Based Registry. *Front Neurol* 2021; **12**: 693017.

6. He A, Merkel B, Brown JWL, et al. Timing of high-efficacy therapy for multiple sclerosis: a retrospective observational cohort study. *Lancet Neurol* 2020; **19**(4): 307-16.

7. Iaffaldano P, Lucisano G, Caputo F, et al. Long-term disability trajectories in relapsing multiple sclerosis patients treated with early intensive or escalation treatment strategies. *Ther Adv Neurol Disord* 2021; **14**: 17562864211019574.

8. Comi G, Filippi M, Barkhof F, et al. Effect of early interferon treatment on conversion to definite multiple sclerosis: a randomised study. *Lancet* 2001; **357**(9268): 1576-82.

9. Giovannoni G, Butzkueven H, Dhib-Jalbut S, et al. Brain health: time matters in multiple sclerosis. *Mult Scler Relat Disord* 2016; **9 Suppl 1**: S5-S48.

10. Ziemssen T, Derfuss T, de Stefano N, et al. Optimizing treatment success in multiple sclerosis. *J Neurol* 2016; **263**(6): 1053-65.

11. Casanova B, Quintanilla-Bordas C, Gascon F. Escalation vs. Early Intense Therapy in Multiple Sclerosis. *J Pers Med* 2022; **12**(1).

12. Spelman T, Magyari M, Piehl F, et al. Treatment Escalation vs Immediate Initiation of Highly Effective Treatment for Patients With Relapsing-Remitting Multiple Sclerosis: Data From 2 Different National Strategies. *JAMA Neurol* 2021; **78**(10): 1197-204.

13. Salas M, Hofman A, Stricker BH. Confounding by indication: an example of variation in the use of epidemiologic terminology. *Am J Epidemiol* 1999; **149**(11): 981-3.

14. Signorello LB, McLaughlin JK, Lipworth L, Friis S, Sorensen HT, Blot WJ. Confounding by indication in epidemiologic studies of commonly used analgesics. *Am J Ther* 2002; **9**(3): 199-205.

15. Akl EA, Briel M, You JJ, et al. Potential impact on estimated treatment effects of information lost to follow-up in randomised controlled trials (LOST-IT): systematic review. *BMJ* 2012; **344**: e2809.

16. Bell ML, Kenward MG, Fairclough DL, Horton NJ. Differential dropout and bias in randomised controlled trials: when it matters and when it may not. *BMJ* 2013; **346**: e8668.

17. Lewin A, Brondeel R, Benmarhnia T, Thomas F, Chaix B. Attrition Bias Related to Missing Outcome Data: A Longitudinal Simulation Study. *Epidemiology* 2018; **29**(1): 87-95.

18. Suissa S. Immortal time bias in observational studies of drug effects. *Pharmacoepidemiol Drug Saf* 2007; **16**(3): 241-9.

19. Levesque LE, Hanley JA, Kezouh A, Suissa S. Problem of immortal time bias in cohort studies: example using statins for preventing progression of diabetes. *BMJ* 2010; **340**: b5087.

20. Goldstein BA, Bhavsar NA, Phelan M, Pencina MJ. Controlling for Informed Presence Bias Due to the Number of Health Encounters in an Electronic Health Record. *Am J Epidemiol* 2016; **184**(11): 847-55.

21. Berkman ND, Santaguida PL, Viswanathan M, Morton SC. The Empirical Evidence of Bias in Trials Measuring Treatment Differences. Rockville (MD); 2014.

22. Greene EJ, Peduzzi P, Dziura J, et al. Estimation of ascertainment bias and its effect on power in clinical trials with time-to-event outcomes. *Stat Med* 2021; **40**(5): 1306-20.

23. Kaufmann M, Puhan MA, Kuhle J, et al. A Framework for Estimating the Burden of Chronic Diseases: Design and Application in the Context of Multiple Sclerosis. *Front Neurol* 2019; **10**: 953.

24. Poser CM, Paty DW, Scheinberg L, et al. New diagnostic criteria for multiple sclerosis: guidelines for research protocols. *Ann Neurol* 1983; **13**(3): 227-31.

25. Polman CH, Reingold SC, Banwell B, et al. Diagnostic criteria for multiple sclerosis: 2010 revisions to the McDonald criteria. *Ann Neurol* 2011; **69**(2): 292-302.

26. Thompson AJ, Banwell BL, Barkhof F, et al. Diagnosis of multiple sclerosis: 2017 revisions of the McDonald criteria. *Lancet Neurol* 2018; **17**(2): 162-73.

27. Sormani MP, Tintore M, Rovaris M, et al. Will Rogers phenomenon in multiple sclerosis. *Ann Neurol* 2008; **64**(4): 428-33.
